# Uncertainty Ordinal Multi-Instance Learning for Breast Cancer Diagnosis

**DOI:** 10.3390/healthcare10112300

**Published:** 2022-11-17

**Authors:** Xinzheng Xu, Qiaoyu Guo, Zhongnian Li, Dechun Li

**Affiliations:** 1 School of Computer Science and Technology, China University of Mining and Technology, Xuzhou 221116, China; 2 Xuzhou Central Hospital, Xuzhou 221116, China

**Keywords:** ordinal classification, multi-instance learning, weak supervision, breast cancer, key instance, uncertainty select

## Abstract

Ordinal multi-instance learning (OMIL) deals with the weak supervision scenario wherein instances in each training bag are not only multi-class but also have rank order relationships between classes, such as breast cancer, which has become one of the most frequent diseases in women. Most of the existing work has generally been to classify the region of interest (mass or microcalcification) on the mammogram as either benign or malignant, while ignoring the normal mammogram classification. Early screening for breast disease is particularly important for further diagnosis. Since early benign lesion areas on a mammogram are very similar to normal tissue, three classifications of mammograms for the improved screening of early benign lesions are necessary. In OMIL, an expert will only label the set of instances (bag), instead of labeling every instance. When labeling efforts are focused on the class of bags, ordinal classes of the instance inside the bag are not labeled. However, recent work on ordinal multi-instance has used the traditional support vector machine to solve the multi-classification problem without utilizing the ordinal information regarding the instances in the bag. In this paper, we propose a method that explicitly models the ordinal class information for bags and instances in bags. Specifically, we specify a key instance from the bag as a positive instance of bags, and design ordinal minimum uncertainty loss to iteratively optimize the selected key instances from the bags. The extensive experimental results clearly prove the effectiveness of the proposed ordinal instance-learning approach, which achieves 52.021% accuracy, 61.471% sensitivity, 47.206% specificity, 57.895% precision, and an 59.629% F1 score on a DDSM dataset.

## 1. Introduction

Breast cancer is one of the most fatal diseases among women. The study of the benign and malignant classifications of breast cancer has been quite extensive. Elmoufidi [1] proposed a framework that uses a modified K-means algorithm to segment the ROI and extract textural features from the ROI for classification. Fahssi et al. [2] presented a novel CAD system for mammography diagnosis. The ROIs are detected by dividing the mammogram into regions and MIL algorithms are applied to identify malignant regions to label the whole mammogram. Most of the existing work has solved the binary classification problem of breast cancer, but there are more normal breast images than abnormal ones. Although the classification of benign and malignant regions of interest in abnormal breast images can achieve good performance, when the classification category becomes normal, benign, or malignant, the classification performance tends to decline. Lamard et al. [3] carried out experiments on binary classification and three-class classification. The experimental results show that the accuracy of the three-class classification is 30% lower than that of the two-class classification, which undoubtedly shows that the three-class classification of breast cancer is more challenging than the two-class classification. Moreover, we found that the classification of normal and benign regions is much more difficult than benign and malignant. The effective screening of early benign lesions is of great importance for further diagnosis and treatment. Since there is a certain order of lesions in the normal, benign, and malignant categories, we transform the general multi-instance learning problem into an orderly multi-instance-learning technique.

Multi-instance learning (MIL) [4,5,6] is a popular learning framework in weakly supervised learning [7,8,9,10]. Different from supervised learning, the data sample for MIL is a bag that consists of a set of instances. In the MIL setting, bags are labeled, while instances in bags are unlabeled. The researchers have studied various paradigms for MIL, including, but not limited to, multiple instance classification(MIC) [11], multiple instance regression, multi-instance clustering, imbalanced multi-instance learning, and multi-instance multi-labeling.

**Figure 1:**
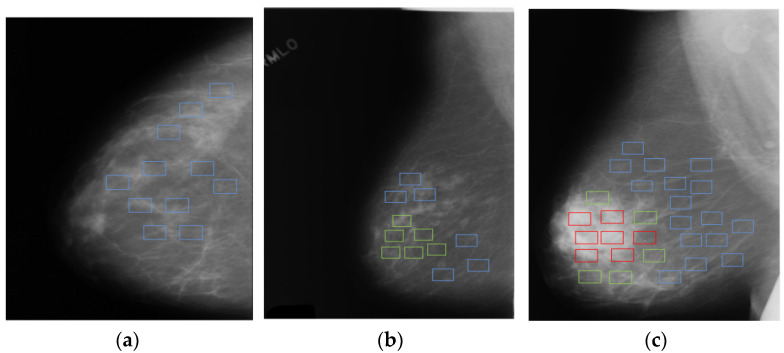
We show images of three types of breast disease lesions: (**a**) normal, (**b**) benign, and (**c**) cancerous. Specifically, the image (bag) consists of several patches (instances). The instances marked by blue rectangles denote normal lesion, green rectangles denote benign lesion, and red rectangles denote cancer. More importantly, the lesion level of the instance in each bag cannot exceed the lesion intensity of the bag.

Here, we consider another typical scenario of MIL-ordinal multiple instance learning [12] (OMIL): the classes of bags are not only ordered, but the instances in the bag are also in a certain rank order. Moreover, the highest level of instance label cannot exceed the label of the bag. For example, in the medical diagnosis of breast cancer, the class of lesion in a mammogram is not only normal, benign, and malignant, but also a rank order among these categories, namely, normal < benign < cancer, as shown in Figure 1. To solve the OMIL problem, previous works have mainly focused on settling one of issues, which could be roughly divided into two problems: one is multiple instance classification, and the other is the ordinal classification problem.

Multi-instance classification is the most widely used MIL paradigm. Zhou et al. [4] proposed a MIEN-metric method that learns discriminative metrics for classifying samples from the observed classes and recognizing the samples from the novel class, which solves the emerging novel class problem concerning muti-instance learning in open and changing complex scenarios. Subsequently, in the study of multi-instance classification, Zhou et al. [5] found that samples in multi-instance learning are described by multiple instances and associated with multiple class labels. Based on a simple degeneration strategy with MIMLBOOST and MIMLSVM algorithms, the proposed multi-instance multi-label framework can deal with problems involving complex objects with multiple semantic meanings. Class imbalance is also a persistent problem in multi-instance classification tasks. Javad et al. [13] proposed a new instance reduction method that preserves the between-class distribution in the balanced data and handles minority class instance reduction in two-class imbalanced data.

The method of multi-instance learning for classification problems generally assumes that the class value is unordered. However, in many practical applications, a natural ordered relationship exists between the categories of instances. Frank et al. [14] proposed a simple method involving the incorporation of a decision tree. Accordingly, the standard classifier algorithm made full use of the ordered information between the class attributes. Different from most of the previous works that employ a loss function based on the absolute difference between the predicted and ground truth class labels, Joan et al. [15] argues that the label values in ordinal classification may be arbitrary, and proposed a network architecture that produces not a single class prediction but an ordered vector.

The recent work by Evan [12] used OMIL in the estimation of ulcerative colitis severity. However, their method transformed the multi-classification problem of OMIL into multiple binary classifiers, which fails to directly model multi-class instance bags and loses the ordered information between classes. To tackle this problem, we propose a discriminative solution to directly model ordinal multi-instance bags, which obviously learns ordered information between classes. Specifically, we exact key instances from the bag as positive instances of bags, and design ordinal minimum uncertainty loss to iteratively optimize the selected key instances from the bags. This study’s main contributions are summarized as follows:

1. We select a key instance as a positive instance of the bags and send the key instance, which has a bag label, to the network for training. The instances selected from the bag at each iteration are uncertain but incur minimal loss.
2. We employ ordinal minimum uncertainty loss to take advantage of the ordered information in classes.
3. We carry out experiments on a DDSM dataset to evaluate the OMIL method. The experimental results demonstrate that our OMIL approach achieves better performance than the existing OMIL method.

The rest of the paper is organized as follows. In Section 2, we introduce some related works about key instances, multi-instance learning, and ordinal classification. The proposed OMIL method and model architecture are proposed in Section 3. In Section 4, the processing of the DDSM dataset and experimental setup are introduced in detail. The experimental results are also presented. In Section 5, in order to verify the effectiveness of our proposed method, we conduct some ablation studies. Finally, Section 6 presents the conclusions of this paper.

## 2. Related Works

This section defines the basis for and reviews the related works on key instances, multi-instance learning, and ordinal classification.

### 2.1. Key Instance

Key instances [16,17,18] play a key role in multi-instance learning, and their labels can trigger the label of the bag. To solve the key instance detection (KID) problem, Liu et al. [16] proposed a voting framework (VF) solution to KID, which utilizes the relationship among instances to form a citer KNN graph, and uses them to define the confidences of the votes of the training instances. However, when encountering a more complex situation, key instance detection may fail. Traditional max pooling cannot make full use of the information from input examples. Yan et al. [19] proposed a novel dynamic-pooling function for MIL that can iteratively update the instance contribution to its bag and highlights the key instance. Inspired by this work, the key instance of our model selected from each bag is constantly optimized. In order to incorporate interpretability into the MIL approach, Ilse et al. [20] proposed an “Attention-based Multiple Instance Learning” method, which pays more attention to positive instances during training. Hence, the attention weights allow us to find a key instance. Notably, this approach makes it clear how each instance contributes to the bag. Nonetheless, shin et al. [17] argued that the performance of the model with respect to key instance detection is limited; that is, an attention-based model [20] focuses on the weight of the positive instance yet the difference between positive and negative instances in the positive bag is not obvious, which may influence performance. To improve the performance of the attention-based model in a KID task, they apply a neural network inversion with a sparseness constraint that updates the instances in a positive bag. In this way, the key instance is better highlighted by using optimized instances.

### 2.2. Multiple Instance Learning

Multi-instance learning [5,6,20,21,22] was proposed by early researchers when studying drug activity prediction. The standard assumption for multi-instance learning is that if a bag is labeled positive, there is at least one positive instance in it. Otherwise, the bag will be labeled negative. The traditional multi-instance hypothesis assumes that the class label of a bag is determined by the key instances in the bag. Multi-instance classification is the most common task in multi-instance learning. Furthermore, it has promoted the emergence of many classification algorithms, such as DD, Citation-KNN, BP-MIP, MI-SVM, mi-SVM, etc. All these MIL algorithms [21] assume that the bag is a binary classification problem, namely, positive and negative bag.

However, in general scenarios, multiple classification tasks are more common than binary classification tasks. Over the last few years, the multi-instance multi-classification task has also attracted the attention of many researchers. Different from ordinary multi-instance learning, which assumes each observation belongs to a class, a framework of multi-instance multi-label learning (MIMIL) can better describe complex objects with several classes. Zhou et al. [5] proposed the MIMLBOOST and MIMLSVM algorithms to solve problems involving complex objects associated with multiple class labels. Following this work, Pham et al. [23] extended the MIMIL problem to the setting wherein a novel class instance is present. They proposed a maximum likelihood method to optimize the model and trained an instance-level classifier for all classes as well as the novel class. Nonetheless, the ground-truth label of the sample is difficult to obtain in the real world. To alleviate this problem, Ishida et al. [24] proposed a novel setting called complementary label learning [24,25] to implement multi-class classification tasks, which only requires the provision of complementary data labels.

In this paper, we focus on the ordinal multi-instance-learning problem. The most similar work to ours is the ordinal multi-instance-learning approach proposed by Evan et al. [12]. One difference is that we directly model the bag as multi-class and select the instance with minimum loss from the bags as a positive instance of the bags to update the model parameters. In this way, each key instance we select from the bag is uncertain and optimal. Nevertheless, they transformed the multi-classification problem of bags into several binary classifiers to deal with, rather than explicitly modeling the bag.

### 2.3. Ordinal Classification

Ordinal classification problems [15,26,27,28] can be viewed as an intermediate problem between classification and regression, where the target variable is both categorical and ordinal. In general classification problems, the categorical variables are taken from a finite set, and there is no metric relationship between the categories, although they are represented numerically. Examples of categorical variables are gender, race, nationality, types of animals, etc. When there is a naturally ordered relationship between categorical variables, the ordinary classification problem transforms into an ordinal classification problem. Some common scenarios include bank credit-rating assessments, determining income level, the lesion grade of cancer, users’ evaluation of service, etc.

Gutierrez et al. [28] assessed the performance of five different methods under different representations of ordinal input variables. The results show that both the Num and NumCDR methods perform well and the Num method directly maps each class to a consecutive natural number. Considering that the misclassification loss of ordinal classification should be different, Beckham et al. [27] proposed a simple modification of the squared error loss, which utilizes the characteristic of the sensitivity of class ordering and guarantees that the possibility of distribution over the classes is discrete. Different from most previous works that compute the absolute difference between the predicted and ground-truth class labels to optimize the loss function, Joan et al. [15] argued that label values in ordinal classification may be arbitrary and replace a single class prediction with an ordered vector.

## 3. Proposed Approach

In this section, we will briefly formalize the OMIL problem and describe several crucial concepts of OMIL. In addition, we introduce a discriminative model for OMIL data wherein the instances in each bag are ordered.

### 3.1. Problem Formalization of OMIL

In the problem definition of OMIL, the training set is represented as: $D=\{(X_{1},y_{1}),\ldots ,(X_{i},y_{i}),(X_{N},y_{N})\},$ where $X_{i}$ denotes a bag, which contains $n_{i}$ instances, and each instance is described by a d-dimensional vector, i.e., $X_{i}=\{x_{i}^{1},x_{i}^{2},\ldots ,x_{i}^{n_{i}}\},x_{i}\in R^{d}$. The labels of the bags are not only multi-class but also have a certain rank order [12] among classes. Simultaneously, the labeling of the instances in the bag cannot exceed the label of the bag. The formal definition is as follows: bag-level class label $y_{i}\in y=\{0,1,\ldots ,C\},(0<1<\cdots <C),$ and instance-level class label $y_{ij}\in y=\{0,1,\cdots ,C_{1}\},(0<1<\cdots <C_{1}\leq y_{i})$. It is supposed that: (1) If a bag $X_{i}$ is labeled as the cth class ⇔ The instance-level label of $X_{i}$ belongs to the set $y^{\prime }=\{0,1,\ldots ,C\}$, (0<1<…<C). The strongest label-level of instances must be the same as the bag label, where the strongest instance-level label is cth class. (2) A label for a bag $X_{i}$ is not assigned to the cth class. ⇔ None of the instances in bag $X_{i}$ belong to the cth class. We give a full description of the three important concepts of OMIL in Table 1.

### 3.2. Model

The proposed model addresses the OMIL problem in two basic steps: (1) after the model outputs the loss of all the instances in the bag, the instance with the least amount of loss is selected as the key instance of the bag. (2) The selected key instances serve as a positive instance of the bag, assign the label of the bag, and participate in the training and optimization of the model.

**Figure 2:**
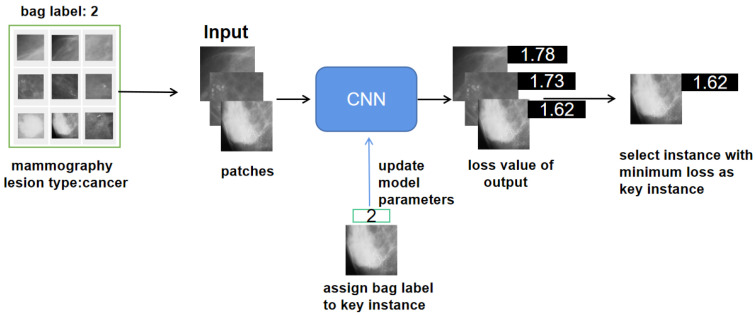
Overall model structure of OOMIL. Mammograms are split into patches, and then fed to the model to select the minimum-loss patch. The key instance-assigned bag labels participate in model training.

Our model is presented in Figure 2. The training sample set is composed of a multi-class bag, which has a class label but the instances in it do not possess label information. The convolutional neural network [29] consists of two convolutional layers with a 5 × 5 and 3 × 3 filter, one pooling layer, and two fully connected layers [30]. The specific structural design of the CNN is shown in Table 2. In the model-training phase, not all instances in a bag participate in training, but the selected key instance [6,17] from the bags can be fed into the network to optimize parameters. In addition, the selected key instances from the bags are not constant, and they will further approach the true positive instances under each optimization of the network.

We illustrate the use of our notation, for example, of OMIL in Table 3, where $Y_{b}\in \arg\max\{0,1,\cdots ,C\}$. This model indicates that the bag label $Y_{b}$ is obtained from the strongest-level label in the instance labels $Y_{b}^{n_{b}}$. Hence, $Y_{b}$ can reveal information about the ordinal class of instances in the bag.

## 4. Experiments

**Figure 3:**
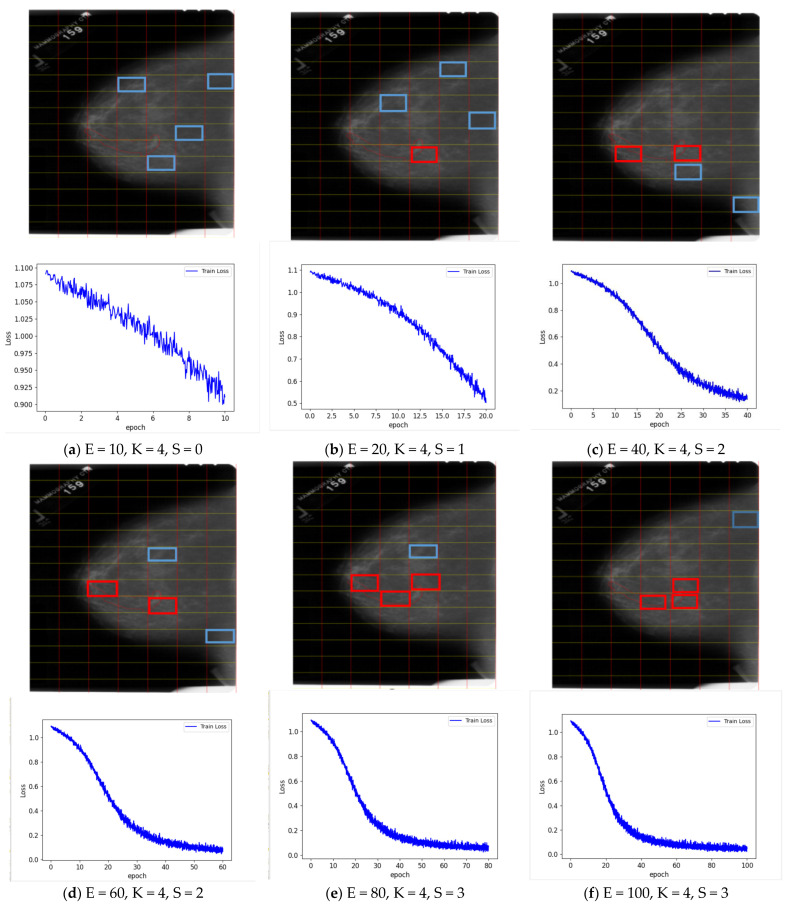
When the key instance K value is 4, the number of positive instances S in the key instances selected by the model and the model training loss change as the epoch E increases E, K, and S represent epoch times, key instance, and positive instance in key instance, respectively. The key instances marked with a blue rectangle denote false instances, whereas the red rectangles represent true instances.

In this section, we evaluate the performance of the proposed novel OOMIL approach against the original OMIL approach, and the results of the comparison when applied to a DDSM dataset are shown in Table 4. Further on, to increase the interpretability of the model, we visualize the process of the model’s selection of key instances from a bag, which is displayed in Figure 3.

### 4.1. Datasets

DDSM [31] is a widely used mammography dataset in computer-aided medical diagnosis. DDSM includes four types of data, namely, normal, benign, benign-without-callback, and cancer, which comprise 10,420 images from 2605 breast cancer cases. For benign and cancer cases, only the images with the lesion area marked by a physician are selected. Hence, our dataset employs 1700 images from normal, benign, and cancer cases. We selected 1360 images from each type of case to form the training set and 340 images to form the test set. 

Some processing operations must be performed on the training set. We first divide each image into grids [32,33,34] with an aspect ratio of 14:7 to obtain 98 patches so that each bag (image) consists of 98 instances (patches). Since some instances come from the noisy region of the image, we use a threshold-processing operation [35,36] to further filter the instances in the bag. To ensure a balanced number of instances per bag, we again expand the dataset with horizontal and vertical flipping as well as rotating image enhancement techniques [37,38], and resize each instance to 224 × 224. Finally, our training set consists of 4080 bags (1360 per lesion type), each containing 70 instances with a 224 × 224 size. To verify the effectiveness of the instance-level classifier trained by our proposed method, we crop the region of interest from each image in the test set as a test example, which has a specific class label.

### 4.2. Experimental Setup

In our experiments, our network is composed of two convolution layers with a 5 × 5 and 3 × 3 filters, a pooling layer with a 2 × 2 kernel, and two fully connected layers [30] with 500 neurons and 1 neuron, respectively. The weights and biases are initialized to be 0. In addition, the k value of the selected key instance is assigned to 4. We employ the approach developed by Adam [39] to optimize our network. Detailed hyper-parameters such as the learning rate, weight decay, eps, and betas are set to 0.0001, 0.0005, and 1 × 10^−6^, (0.9, 0.99). We choose pytorch as the deep-learning framework [40] and write the code in python. The experiments are run on a PC with AMD EPYC-7302 CPU and 64 GB RAM.

### 4.3. Empirical Results of OMIL

Here, we present the comparison results for our novel OOMIL method and existing OMIL method. The experimental results in Table 4 show that our method performs better than the OMIL when applied to the DDSM dataset. In order to intuitively show the effectiveness of our proposed method, we visualize the process of the model selecting the key instances from a bag. The process is shown in Figure 3. The letter E represents the training epoch of the model, K represents the number of key instances to pick from the bag, and S denotes the number of key instances that represent positive instances. Instances with a blue rectangle represent the key instances that are not selected correctly. On the contrary, the red rectangles represent key instances that are selected correctly. As is vividly shown in Figure 3, when the training epoch increases, the number of real key instances that are selected out also increases. Thus, the effectiveness of our proposed method has been proven.

## 5. Ablation Study and Discussion

In this section, we conduct some ablation studies regarding the effect of the k value of the key instance on each class prediction and different loss functions across the DDSM dataset. In addition, the evaluation metrics of the model are presented in Table 5.

***Each class accuracy on different k values of the key instances.*** As mentioned in Section 3, a key instance [16,18] is an instance with minimum loss, which is closest to the positive instance in the bag. The accuracy of the key instance’s selection further affects the effectiveness of the model’s ability to learn. To reduce the probability of the erroneous selection of key instances, we select the first k instances with minimum loss as the key instances of the bag. Hence, we carry out comparison experiments to study the influence of different k values. In Table 6, we show each class result of the different k values on the DDSM dataset. In addition, we can see that as the number of key instances selected increases, each category has a more even prediction probability instead of being inclined towards selecting a certain class. That is, the model performs well overall.

***Different loss functions’ accuracies on different k values of key instance.*** Through previous MIL tasks [4,23], cross entropy loss [41] has proven to be one of the most commonly used loss functions in MIL methods. Instead, our OMIL uses the ordinal minimum uncertainty loss during training. To discuss the influence of different loss functions, we conduct ample comparative experiments on cross entropy loss and minimum uncertainty ordinal loss. In order to apply the Cross Entropy loss to our OMIL, we change the network architecture by replacing the last fully connected layer, which has one neuron, with a fully connected layer, which has three neurons and employs a SoftMax function. Then, combining the bag label with the instance probability, we can calculate the Cross Entropy loss. Finally, we can obtain the loss of each instance from the bag and obtain the instance with minimum loss. Table 7 indicates that, with respect to the DDSM dataset, the results based on the minimum uncertainty ordinal loss are better than cross entropy loss. This proves the effectiveness of the minimum uncertainty ordinal loss to our OMIL.

The experimental results in Table 7 show that the accuracy of the model does not always rise with the number of key instances (K). A possible reason is that the sample imbalance affects the improvement of the model’s performance. In our training bags, the number of normal instances is much greater than that of benign and cancer instances, which is due to the limitations of the mammogram itself. The lesion regions in the mammogram may account for only 4% of the whole image. The model performs well and the prediction accuracy for each class is relatively average when the value of K is 4. The model tends to predict class 0 in the case of K being 5. The reason is that it is difficult to pick the correct instances in the early stage of model training, and the model’s fault tolerance rate is low if the number of key instances is too small. However, when the number of key instances is too large, false key instances are far larger in number than true key instances, which also leads to the degradation of the model’s performance.

Currently, most of the existing work on breast cancer classification addresses the binary classification problem, that is, judging whether the ROI (mass or calcification) is benign or malignant. Elmoufidi [1] proposed a framework comprising two steps of ROI segmentation and feature extraction from ROI for classification. The experimental results regarding the method’s sensitivity, specificity, and accuracy are 94.46%, 94.40%, and 94.43%. Fahssi et al. [2] presented a novel CAD system for mammography diagnosis. They first partition the mammogram into regions and detect the ROI, and then use MIL algorithms to identify malignant regions in order to assign the label to the whole mammogram. This achieves 90.84% sensitivity, 90.17% specificity, and 90.33% accuracy. However, in practical situations, normal cases are generally more frequent than abnormal cases in the diagnosis of breast disease. There is no doubt that screening for abnormal mammograms from large numbers of normal mammograms is energy-consuming work for physicians. Accordingly, the classifier we trained was three-class, classifying mammograms as normal, benign, or malignant. The three-class task is more challenging than ordinary binary classification, as the instance features of normal and benign categories are very similar, especially with respect to benign lesions in early breast cancer. Lamard et al. [3] carried out experiments on binary classification, which achieved an accuracy of 91.1%, while the accuracy of the three-class classification was only 62.1%, a drop of almost 30%, which strongly suggests that three-class classification tasks are more difficult than binary classification tasks. Although our experiment achieved only 52.021% accuracy, to the best of our knowledge, we are the first to propose a novel OMIL method to directly model multi-category bags rather than transforming them into multiple binary classifiers for multi-categories. During the experiment, we found that the accuracy of malignant classification is generally higher than normal and benign, while the accuracy of normal and benign categories is similar. How to more effectively classify normal and benign mammograms and improve the accuracy of model prediction is the key point to consider in our next experiment.

## 6. Conclusions

In this paper, we addressed the ordinal multi-instance-learning problem in breast cancer diagnosis using mammograms. Compared with the binary classification of mammograms, the three-classification task provides superior support for the screening of early breast cancer, as the features of normal tissues and benign lesion areas are very similar. During the experiment, we found that the predictive accuracy with respect to the malignant categories is generally higher than that of the normal and benign categories. Simultaneously, the accuracy gap between the normal and benign categories is small. This also shows that benign lesions can easily be predicted as normal categories, which is very influential in preventing further deterioration in early breast cancer. Hence, we propose a method that directly models the ordinal class information for bags and the instances in the bags. Moreover, to ensure that the key instance selected in the bag is closer to the real positive instances, we employed minimum uncertainty ordinal loss to iteratively optimize the selected key instances from the bags. To increase the interpretability of the model, the simple grid segmentation method acted as the generator of bags, which allowed for the convenient recording of the specific location of instances in the bag to visualize the process of the model’s instance selection. Other more elaborate bag generation methods such as SBN, ImaBag, BlobBag, etc., may improve the performance of the model. The problem of sample imbalance is also not addressed by our current work. The number of normal samples is much higher than benign and cancer samples, leading to the higher prediction accuracy of the model for normal instances. A model with strong generalizability is trained by introducing cost-sensitive methods that impose different penalties for different numbers of samples, which is the focus of our future work. Overall, our method breaks down the barrier that medical images are difficult to label and supply help for breast cancer diagnosis in the future.

## Figures and Tables

**Table 1:** Three relevant, important concepts of OMIL.

Concepts	Descriptions
Bags	The data unit of OMIL dataset that has labels. Each bag contains several ordinal instances without label information.
Instance	The label class of instances in a bag is ordered, and the largest category belongs to the bag category.
Key instance	The instance with minimum loss, which is selected from bag $X_{i}$. Bag-level label is provided for the instance to participate in the model-training process.

**Table 2:** Setting of the parameters of convolutional neural network (CNN) in the model structure diagram.

Network Layer	Input	Output	Filter	Stride	Padding	Parameter
Input	224 × 224 × 1	224 × 224 × 1	-	-	-	0
Conv1	224 × 224 × 1	220 × 220 × 10	5 × 5 × 10	1	0	260
Relu	220 × 220 × 10	220 × 220 × 10	-	-	-	0
MaxPool	220 × 220 × 10	110 × 110 × 10	2 × 2	2	0	0
Conv2	110 × 110 × 10	108 × 108 × 20	3 × 3 × 20	1	0	1820
Relu	108 × 108 × 20	108 × 108 × 20	-	-	-	0
FC1	108 × 108 × 20	500	-	-	-	116,640,500
FC2	500	1	-	-	-	501

**Table 3:** Instance labels and bag labels of the proposed model.

Bag 1	Bag 2	Bag 3
$n_{1}$ = 1	$n_{2}$ = 2	$n_{3}$ = 3
$y_{11}$ = normal $y_{12}$ = normal ⋮ $y_{1n}$ = normal	$y_{21}$ = normal $y_{22}$ = normal ⋮ $y_{2n}$ = benign	$y_{31}$ = normal $y_{32}$ = benign ⋮ $y_{3n}$ = cancer
$Y_{1}^{1}$ = {normal}	$Y_{2}^{2}$ = {normal, benign}	$Y_{3}^{3}$ = {normal, benign, cancer}
$Y_{1}$ = {normal}	$Y_{2}$ = {benign}	$Y_{3}$ = {cancer}

**Table 4:** Test accuracy comparison results for OMIL and OOMIL (our) method.

Dataset	OMIL	OOMIL (Our Method)
DDSM	49.882	52.021

**Table 5:** Different performance measurements of our model.

Proposed Method	Sensitivity (%)	Specificity (%)	Precision (%)	F1 (%)
OOMIL	61.471	47.206	57.895	59.629

**Table 6:** Class accuracy comparison of number set K for key instances in the model. K takes values from 1 to 5, which denote different values of selected key instances.

Class	K				
	1	2	3	4	5
0	20.294	43.529	57.059	52.059	71.765
1	26.176	32.941	22.059	42.353	35.882
2	85.294	54.706	60.000	49.118	22.353

**Table 7:** Average test accuracy comparison of different loss functions on number set K for key instances. K ranges from 1 to 5.

Loss	K				
	1	2	3	4	5
cross entropy (OOMIL)	47.255	47.059	49.706	51.176	47.667
min-uncertainty ordinal (OOMIL)	48.529	47.451	49.679	52.021	47.983
cross entropy (OMIL)	46.972	47.183	48.127	48.743	46.430
min-uncertainty ordinal (OMIL)	47.943	47.372	49.232	49.882	46.846

## Data Availability

Not applicable.
